# It can be unnecessary to combine common synovial fluid analysis and alpha-defensin tests for periprosthetic joint infection diagnosis

**DOI:** 10.1186/s12891-023-06594-5

**Published:** 2023-06-29

**Authors:** Hao Li, Rui Li, Niu Erlong, Wei Chai, LiBo Hao, Chi Xu, Jun Fu, Jiying Chen, Fangzheng Zhu

**Affiliations:** 1 Medical School of Chinese PLA, Beijing, People’s Republic of China; 2 Department of Orthopedic Surgery, The First Medical Center, Chinese PLA General Hospital, 28 Fuxing Road, Beijing, People’s Republic of China; 3 Senior Department of Orthopedics, Fourth Medical Center of PLA General Hospital, Beijing, People’s Republic of China; 4 Department of Orthopedics, 305 Hospital of PLA, Beijing, People’s Republic of China; 5 Department of Orthopaedics, PLA Rocket Force Characteristics Medical Center, Beijing, 100088 China

**Keywords:** Periprosthetic joint infection, The 2018 ICM criteria, Alpha-defensin, Total joint arthroplasty

## Abstract

**Background:**

Periprosthetic joint infection is a serious complication after total joint arthroplasty. Despite that alpha-defensin was used as diagnostic test in the 2018 ICM (international consensus meeting) criteria, its position in the PJI diagnostic pipeline was controversial. Therefore, we performed a retrospective pilot study to identify whether synovial fluid alpha-defensin test was necessary when corresponding synovial fluid analysis (WBC count, PMN% and LE tests) was performed.

**Methods:**

Between May 2015 and October 2018, a total of 90 suspected PJI patients who underwent revisions after TJA were included in this study. Based on the 2018 ICM criteria, the interobserver agreements between preoperative diagnostic results and postoperative diagnostic results and the interobserver reliability between preoperative diagnostic results and postoperative diagnostic results with or without synovial fluid alpha-defensin tests were calculated. After that, the ROC analysis, and the direct cost-effectiveness of adding alpha-defensin was performed.

**Results:**

There were 48,16 and 26 patients in the PJI group, inconclusive group and non-PJI group, respectively. Adding the alpha-defensin tests into 2018 ICM criteria can’t change the preoperative diagnostic results, postoperative diagnostic results, and the concordance between preoperative and postoperative diagnostic results. Moreover, the Risk–benefit Ratio is over 90 per changed decision and the direct cost-effectiveness of alpha-defensin was more than $8370($93*90) per case.

**Conclusions:**

Alpha-defensin assay exhibit high sensitivity and specificity for PJI detection as a standalone test based on the 2018 ICM criteria. However, the additional order of Alpha-defensin can’t offer additional evidence for PJI diagnosis when corresponding synovial fluid analysis was performed (synovial fluid WBC count, PMN% and LE strip tests).

**Evidence level:**

Level II, Diagnostic study.

## Background

Periprosthetic joint infection is a serious complication after total joint arthroplasty. However, its diagnosis remains controversial and challenging [1, 2]. In recent years, several workgroups have established different criteria for PJI diagnosis. In 2011, MSIS criteria was built and it played a pivotal role in PJI diagnosis with subsequent revision type in 2014 [3]. In 2013, IDSA criteria was established by Infection Disease society of America [4]. Besides, new ICM criteria was constructed in 2018 [5]. In 2020, a new EBJIS criteria was built by European Bone and Joint Infection Society [6].

Alpha-defensin is an antimicrobial peptide released by neutrophils in the presence of various pathogens so the levels of synovial fluid Alpha-defensin can increase with development of PJI and it has the potential feature to be a biomarker of infection. Recently, several studies revealed that Alpha-defensin (AD) showed excellent PJI diagnostic value and it was adopted by the some established PJI definition criteria. However, despite alpha-defensin was included in the 2018 ICM criteria, it may not suit in routinely clinical practice because some studies suggested that alpha-defensin perform similar diagnostic value compared to synovial fluid analysis (synovial fluid WBC count, polymorphonuclear percentage (PMN%) and leukocyte esterase (LE) strip test) [7–12].

In the 2018 ICM criteria, positive synovial fluid alpha-defensin, WBC count and LE strip tests were scored 3 points but whether all these three tests should be ordered simultaneously or partly remain unknown and controversial [5]. Some studies revealed that alpha-defensin performed similar diagnostic value compared to synovial fluid WBC count, PMN% and LE tests but the cost of latter ones was significantly lower than the former one [7, 9, 11]. Thus, these studies questioned the routine clinical use of alpha-defensin in PJI diagnosis [7, 13–15]. Considering the costs and the controversial diagnostic value of alpha-defensin tests mentioned above, when to perform synovial fluid defensin test and how to integrate alpha-defensin into common PJI diagnosis was controversial based on current criteria [5].

Therefore, to address the questions mentioned above and clarify the diagnostic value of alpha-defensin in current 2018 ICM criteria, we performed a retrospective cohort study to identify whether synovial fluid alpha-defensin tests was necessary when corresponding synovial fluid analysis (WBC count, PMN% and LE tests) was performed based on the 2018 ICM criteria. We hypothesize that the combination of synovial fluid analysis has similar sensitivity and specificity than the combination of synovial fluid WBC and alpha defensin in PJI diagnosis.

## Materials and methods

Between May 2015 and October 2018, a total of 90 suspected PJI patients after TJA were included in this study finally. Based on the institutional protocol, suspected PJI was considered when one of the following criteria was met after TJA.

1. Acute or persistent rest pain, swelling, redness or warmth around the joints,
2. Elevated erythrocyte sedimentation rate (ESR) or C-reactive protein (CRP) level:
3. ESR > 30 mm/hr, CRP > 10 mg/L (with > 6 weeks of symptoms)
4. CRP > 100 mg/L (with < 6 weeks of symptoms),
5. Implant failure within 5 years after total joint arthroplasty such as implant mechanical failure and loosening.

Once PJI was suspected, the joint aspiration was performed by two experienced surgeons (the first and second authors) [16]. It was feasible to use synovial fluid for aerobic, anaerobic, WBC (count and differential), LE test and AD test if more than 1.5 mL synovial fluid was obtained. The obtained synovial fluid was injected into a BacT/ALERT FA FAN (fastidious antimicrobial neutralization) (bioMerieux) bottle (≥ 0.5 ml) for anaerobic bacterial culture and a BacT/ALERT PF Pediatric FAN (bioMerieux) bottle (≥ 0.5 ml) for aerobic bacterial and fungal culture, respectively. Each bottle was incubated for 2 weeks, and VITEK-MS (bioMerieux) was used for microorganism identification if pathogens were detected. Besides, synovial fluid (≥ 200ul) was sent for WBC analysis (count and PMN%). One drop of synovial fluid (10ul) was immediately applied to a LE test strip (Aution Sticks 10PA, Arkray, Japan). The LE strip test result was read based on changes in the strip pad color approximately three minutes later. Five different color grades are shown on the color chart (neg, ±, + , 2 + , and 3 +). In the present study, we used 2 + as the positive threshold. Then the residual synovial fluid was stored at -80℃ for following alpha-defensin tests.

### Synovial fluid Alpha-defensin test and the identification of cut-off

The stored synovial fluid was tested for alpha-defensin (human alpha-defensin 1, R&D, USA) by standard enzyme-linked immunosorbent assay (ELISA) after thawing. Considering the alpha-defensin was interpreted into the 2018 ICM criteria and reducing potentially bias, the 2011 MSIS criteria were used as the “PJI reference standard” when the optimal cut-off of alpha-defensin ELISA tests was identified.

### Chart review and the diagnostic criteria

In this study, the 2018 ICM criteria were used as the “reference standard” for PJI diagnosis. The diagnosis of PJI were performed preoperatively and intraoperatively. Hence, for a single case, PJI diagnosis results were divided into preoperative diagnosis results and intraoperative diagnosis results. And for each result, the case was classified into “PJI”, “non-PJI” and “inconclusive”.

In order to evaluate the utility of Alpha-defensin in PJI diagnosis, the 2018 ICM criteria were applied on each case 4 times preoperatively and postoperatively, respectively. In the primary evaluation of synovial fluid tests, only the synovial fluid WBC count were taken into the calculation of total preoperative and postoperative scores based on the 2018 ICM criteria (Rater A). Then, both the synovial fluid WBC count and Alpha-defensin tests were taken into calculation (Rater B). After that, the synovial fluid WBC count, LE strip test and Alpha-defensin test were all taken into calculation (Rater D). Besides, we also calculated the total preoperative and postoperative scores by taking the WBC count and LE strip tests (Rater C).

### The utility of synovial fluid alpha-defensin

To identify the utility of synovial fluid alpha-defensin test in PJI diagnosis, the new diagnostic criteria was modified from the primary ones after removing the criterion about synovial fluid alpha-defensin test. Then, the concordance between the primary ones and the modified ones were compared.

Inclusion criteria:

1. There was still enough synovial fluid for alpha-defensin tests after performing synovial fluid WBC, PMN%, LE strip tests and cultures.
2. Patients underwent revisions in the institution.
3. Chronic phase patients whose symptoms lasts for more than 3 months.

Exclusion criteria:

1. Dry joints
2. Not enough synovial fluid to conducting alpha-defensin tests after performing (synovial fluid WBC, PMN%, LE strip tests and cultures)
3. Unreadable LE strip tests.
4. Acute phase patients whose symptoms lasts for less than 3 months.
5. Patients whose Joint revisions were performed in other institutions and patients without Joint revisions.

### Statistical analysis

The variables were divided into continuous variables and dichotomous data based on the types of data. A normal distribution test was used to evaluate the distribution of continuous variables. The continuous variables were described as means if the normal distribution was achieved. Otherwise, corresponding medians were calculated. Rand sum test and ANOVA were used to detect the difference if the corresponding applicable conditions were met. Dichotomous data were described as frequencies and compared by chi-squared test subsequently.

In this study, the 2018 ICM criteria were used as the “reference standard” for PJI diagnosis. We used Cohen’s kappa [17] to examine the intraclass correlation coefficient (ICC) between the diagnostic results with alpha-defensin and the diagnostic results without alpha-defensin preoperatively and postoperatively, respectively. Besides, Cohen’s kappa was also used to examine the intraclass correlation coefficient (ICC) between preoperative diagnostic results and postoperative diagnostic results with or without synovial fluid alpha-defensin tests.

To further evaluate the sensitivity, specificity, positive predictive value (PPV) and negative predictive value (NPV) of the synovial fluid test (synovial fluid alpha-defensin, LE strip tests, WBC count and PMN%), 3 models were built based on the postoperative diagnosis result of 2018 ICM criteria. In model 1, the “inconclusive cases” were excluded from the cohort when these diagnostic indexes were calculated. In model 2, the “inconclusive cases” were added into the “PJI group”. And in model 3, the “inconclusive cases” were added into the “non-PJI group” to perform analysis. Furthermore, the Receiver Operator Curve (ROC) analysis and the Area Under the Curve (AUC) was also performed in these three models.

The risk–benefit ratio (RBR) was calculated based on how many diagnostic results changed when alpha-defensin tests were added into the diagnostic panel. Thus, a formula was expressed as: RBR = The number of patients whose alpha-defensin tests don’t change the diagnostic results/The number of patients whose alpha-defensin change the diagnostic results. Moreover, Direct cost-effectiveness of adding alpha-defensin to diagnostic panel was calculated according to following formula: Direct effectiveness = (RBR + 1) * the cost of per alpha-defensin test.

In this study, SPSS (IBM, version: 26.0), RStudio (version: 1.4.1717) and R (version: 4.1.1) were used to process data. *P* < 0.05 indicates statistical significance.

## Results

### The demographic characteristics of patients included in this study

**Fig. 1:**
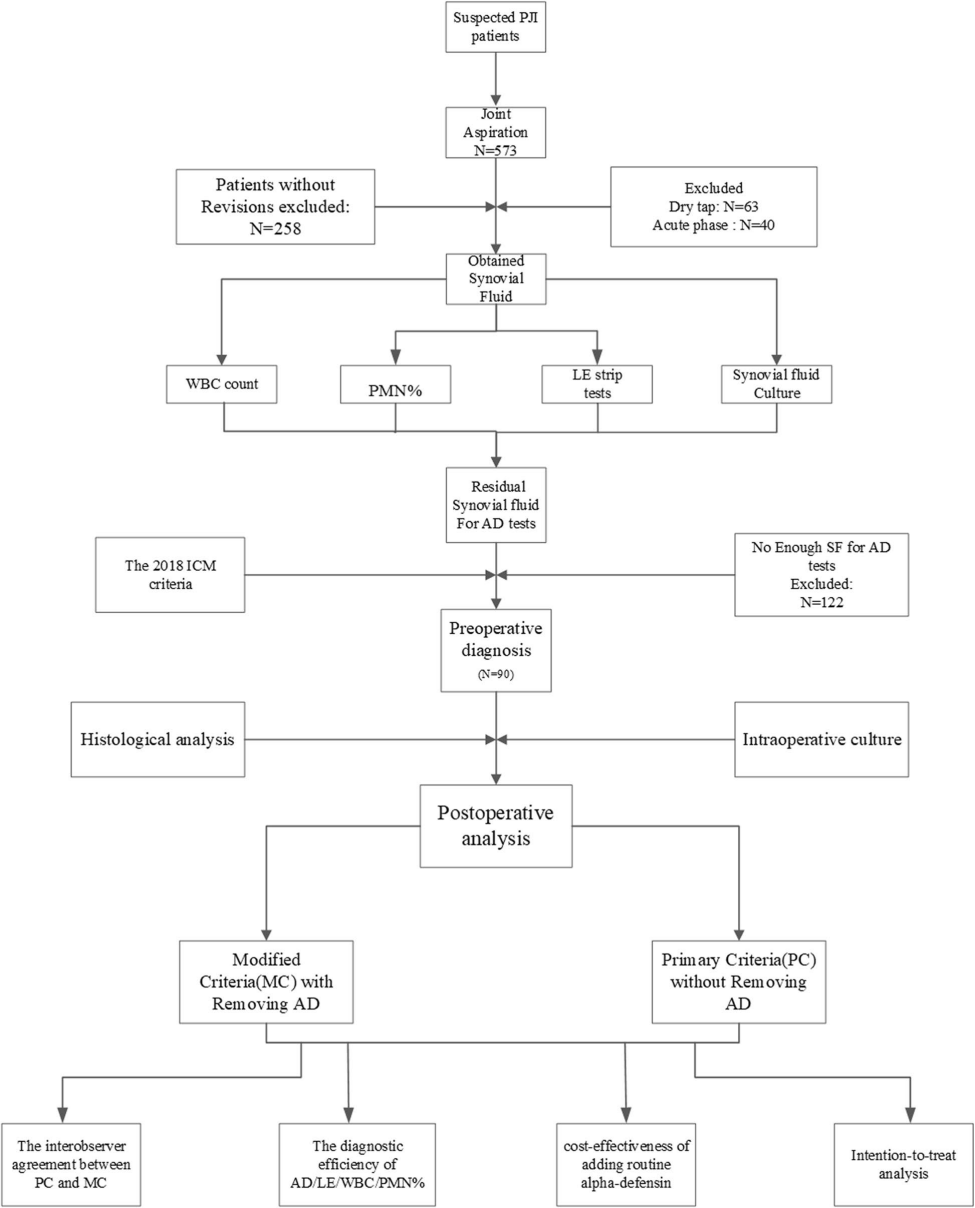
The study design and the patients included in the study

There were 90 patients included in this study (Fig. 1). And based on the 2018 ICM criteria, there were 48,16 and 26 patients in the PJI group, inconclusive group and non-PJI group, respectively. The median age in this PJI group, inconclusive cases and Non-PJI group and were 66 years, 63 years and 68 years, respectively. The median BMI in this PJI group, inconclusive cases and Non-PJI group and were 25.35kg/m^2^, 23.81kg/m^2^ and 26.6kg/m^2^, respectively. The details about the demographic characteristics of patients included in this study was summarized in Table 1.

**Table 1:** The demographic characteristics of patients included in this study

	^a^PJI patients (*N* = 48)	Inconclusive cases (*N* = 16)	^a^Non-PJI patients (*N* = 26)	*P* values:
**Age **^b^** (years)**	66(39,84)	63(53,81)	68 (44,87)	0.770
**Male**	27,56.25%	3,18.75%	7,26.92%	0.12
**Height **^b^** (cm)**	165(146,184)	160(150,169)	160(149,180)	0.009
**Weight **^b^** (Kg)**	68(50,95)	65(64.5,72)	69.5(50,105)	0.097
**BMI **^b^** (kg/m **^2^**)**	25.35(17.30,36.49)	23.81(17.5,31.11)	26.6(19.53,32.41)	0.064
**Knee n, %**	30,62.5%	9,56.25%	14,53.85%	0.749
**Inflammatory Joint Diseases n, %**	1,2.08%	1,6.25%	6,23.08%	0.394
**ESR **^b^** (mm/hr)**	56(7,95)	13(1,88)	11.5(3,27)	< 0.0001
**CRP **^b^** (mg/dL)**	19(0.5,257)	3.21(1,40.5)	1.02(0.5,27.1)	< 0.0001
**WBC Count** ^b^	16,000(7,540,000)	610(10,15,300)	190(0,2400)	< 0.0001
** > 3000 n,%**	40,83.33%	2,12.5%	0,0	< 0.0001
** < 3000 n,%**	8,16.67%	14,87.5%	26,100%	< 0.0001
**PMN%** ^b^	0.9(0.05,0.98)	0.35(0.05,0.95)	0.2(0.05,0.95)	< 0.0001
** < 70 n,%**	7,14.58%	10,62.5%	17,65.39	< 0.0001
** > 70 n,%**	41,85.42%	6,37.5%	9,34.6%	< 0.0001
LE strip test				
** Negative n,%**	9,18.75%	13,81.25%	23,88.46%	< 0.0001
** Positive n,%**	39,81.25%	3,18.75%	3,11.54%	< 0.0001
**Alpha-defensin (μg/mL)**^b^	162.51(0.863,1433.61)	1.038(0.036,141.38)	0.770(0.049,6.86)	< 0.0001
** Positive Histological Analysis(> 5/HP) n,%**	32,66.67%	8,50%	0	< 0.0001
** The Presence of Sinus n,%**	2,4.17%	0	0	0.279
** Single Positive Culture n,%**	0	16,100%	0	< 0.0001
** Two identical Positive Cultures n,%**	27,56.25%	0	0	< 0.0001

^a^The PJI group, non-PJI group and inconclusive cases were identified by the postoperative diagnostic results based on the 2018 ICM criteria

bThe values were given as medians and the range in the parentheses

### Preoperative and Postoperative intraclass correlation coefficient (ICC) about the presence of a PJI based on 2018 ICM PJI definition

For preoperative data with or without alpha-defensin, all three raters (rater A, rater B and rater C) had excellent ICC (0.85-1) compared to the combination of three synovial fluid tests (WBC count, Alpha-defensin test and LE strip test). And the combination of WBC count and LE strip test has identical diagnostic results compared to the combination of these three synovial fluid tests (ICC: 1) based on the 2018 ICM criteria (Table 2).

**Table 2:** Intraclass correlation coefficient (ICC) between the preoperative and postoperative diagnosis of prosthetic joint infection using the 2018 ICM criteria

**Variables**	**Preoperative diagnosis (95%CI)**	**Postoperative Diagnosis (95%CI)**
***ICC***		
Rater A and Rater D	0.85(0.76,0.94)	0.792(0.68,0.90)
Rater B and Rater D	1(1,1)	1(1,1)
Rater C and Rater D	0.869(0.78,0.96)	0.809(0.70,0.91)
Rater A and Rater C	0.981(0.94,1)	0.982(0.94,1)
Rater A and Rater B	0.850(0.76,0.94)	0.792(0.68,0.90)
***ICC***	***Preoperative and Postoperative Diagnosis (95%CI)***	
Rater A	0.779(0.67,0.89)	
Rater B	0.733(0.61,0.85)	
Rater C	0.778(0.67,0.89)	
Rater D	0.733(0.61,0.85)	

Rater A: Synovial fluid WBC count; Rater B: The Combination of Synovial fluid WBC count and LE strip test; Rater C: The Combination of Synovial fluid WBC count and alpha-defensin tests; Rater D: The Combination of Synovial fluid WBC count, LE strip tests and alpha-defensin tests

For postoperative data with or without alpha-defensin, all three raters had excellent ICC (0.792-1) compared to the combination of three synovial fluid tests (WBC count, Alpha-defensin test and LE strip test). And the combination of WBC count and LE strip test also has identical diagnostic results compared to the combination of three synovial fluid tests (ICC: 1) based on the 2018 ICM criteria (Table 2). And the details about the change of preoperative and postoperative diagnostic results when adding alpha-defensin into diagnostic panel was summarized in Table 3. Generally, no more than 1 diagnostic result changed when additional alpha-defensin was ordered based on the 2018 ICM criteria.

**Table 3:** Change in periprosthetic joint infection diagnosis with or without AD test results

^**a**^ **Prior to surgery:**			
***WBC***	***WBC*** ** + ** ***AD***		
	PJI	Inconclusive	non-PJI
PJI	45	0	0
Inconclusive	0	9	0
Non-PJI	0	1	35
^**a**^ **After revision:**			
***WBC***	***WBC*** ** + ** ***AD***		
	PJI	Inconclusive	non-PJI
PJI	47	0	0
Inconclusive	1	16	0
Non-PJI	0	0	26
^**b**^ **Prior to surgery:**			
***WBC*** ** + ** ***LE***	***WBC*** ** + ** ***AD*** ** + ** ***LE***		
	PJI	Inconclusive	non-PJI
PJI	47	0	0
Inconclusive	0	13	0
Non-PJI	0	0	30
^**b**^ **After revision:**			
***WBC*** ** + ** ***LE***	***WBC*** ** + ** ***AD*** ** + ** ***LE***		
	PJI	Inconclusive	non-PJI
PJI	54	0	0
Inconclusive	0	14	0
Non-PJI	0	0	22

^a^*WBC vs WBC + AD*

^b^*WBC + LE vs WBC + AD + LE*

### The intraclass correlation coefficient (ICC) between Preoperative Diagnosis Results and Postoperative Diagnostic Results

For preoperative data and postoperative data, all 4 raters (rater A, rater B, rater C and rater D) had substantial ICC (0.733-0.779) between preoperative diagnosis and postoperative diagnosis (Table 2) based on the 2018 ICM criteria. And the raters with LE strip tests had lower ICC compared to that without LE strip test (rater A and rater C).

### The Risk–benefit Ratio and Direct Cost-effectiveness of Adding Alpha-defensin into PJI Diagnostic Panel

Compared to the single performance of synovial WBC test, additional 10.25 and 11 orders of LE strip test can change one preoperative and postoperative diagnosis classification based on 2018 ICM criteria, respectively. Compared to the performance of the combination of synovial fluid WBC tests and LE test, additional order of alpha-defensin can’t change the preoperative and postoperative diagnostic classification based on the 2018 ICM criteria. Compared to the performance of the combination of synovial fluid WBC tests and alpha-defensin test, additional 11.85 and 8 orders of LE strip tests can change one preoperative and postoperative diagnostic classification based on the 2018 ICM criteria, respectively.

There were 1 preoperative and postoperative case in which the PJI diagnosis results changed because of Alpha-defensin test based on the 2018 ICM criteria when alpha-defensin was added into the primary WBC count test. The cost-effectiveness of using Alpha-defensin was $8370 ($93 per test * 90 patients) per changed decision before surgery and $8370 ($93 per test * 90 patients) per changed decision after surgery in this situation. There was no preoperative and postoperative case in which the PJI diagnosis results changed because of Alpha-defensin test when orthogonal WBC count test and LE strip test were performed. The cost-effectiveness of using Alpha-defensin was $8370 with no changed decision before surgery and after surgery. The details about the cost-effectiveness were summarized in Table 4.

**Table 4:** The risk–benefit ratio and direct cost-effectiveness of adding alpha-defensin or LE test into PJI diagnostic panel

*Group*	*Preoperative*		*Postoperative*	
	***risk–benefit ratio (RBR)*** ^a^	***Direct*** ***Cost-effectiveness*** ^b^	***risk–benefit ratio (RBR)***	***Direct*** ***Cost-effectiveness***
*WBC vs (WBC* + *LE)*	10.25	$112.5	79/11,7.18	$81.8
*(WBC* + *LE) vs (WBC* + *LE* + *AD)*	NA	> $8370	90/0, NA	> $8370
*WBC vs (WBC* + *AD)*	89	$8370	89/1,89	$8370
*(WBC* + *AD) vs (WBC* + *AD* + *LE)*	11.85	$128.5	80/10,8	$90

*WBC* Synovial fluid white blood cell count, *AD* Synovial fluid alpha-defensin tests. ($93 per test), *LE* LE strip tests. ($10 per test)

^a^RBR = The number of patients whose alpha-defensin tests don’t change the diagnostic results/The number of patients whose alpha-defensin change the diagnostic results

^b^Direct cost-effectiveness: Direct effectiveness = (RBR + 1) * the cost of per alpha-defensin test

### Correlation between synovial fluid analysis tests and PJI diagnosis results

**Fig. 2:**
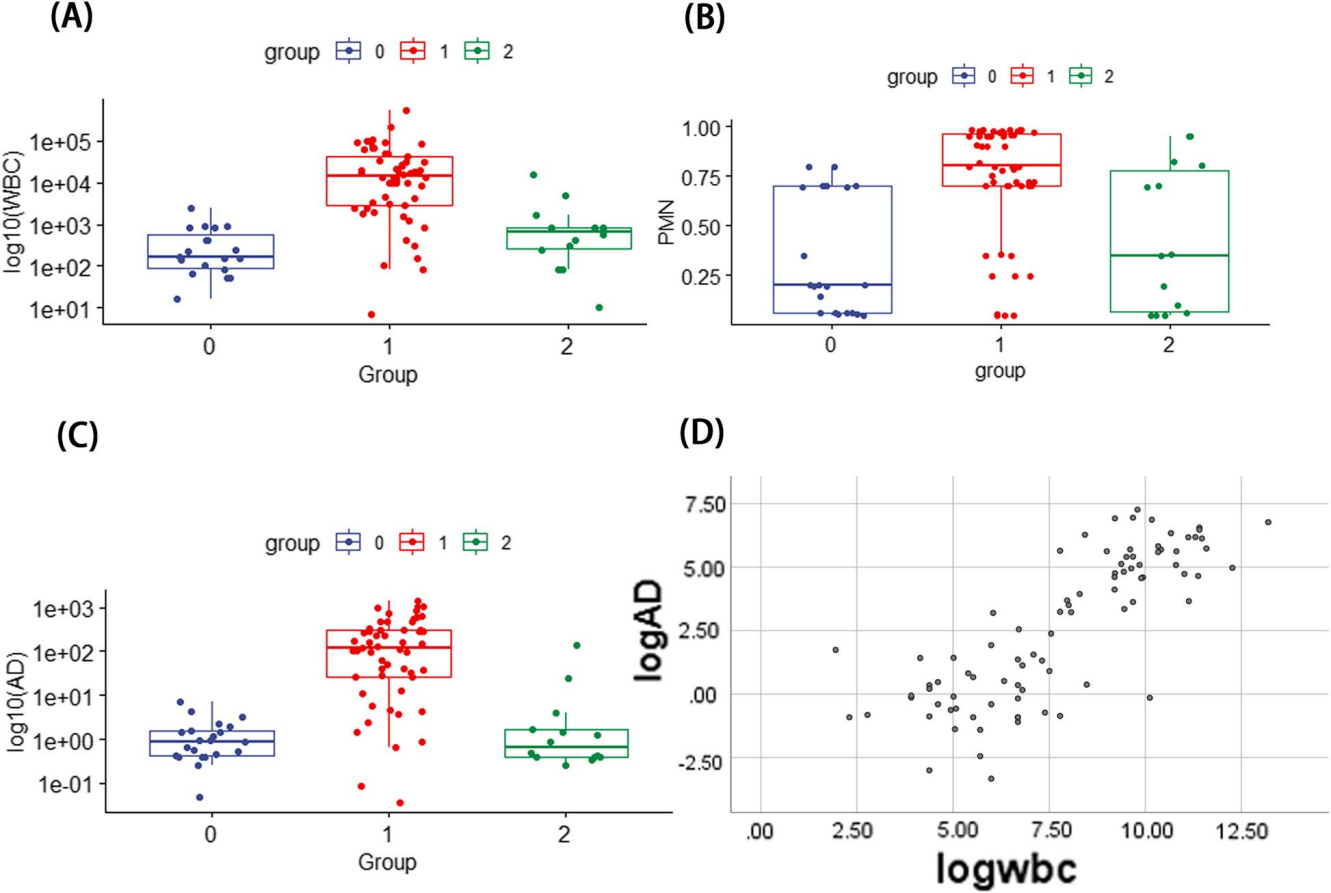
**A** The boxplot showing the relationship between synovial fluid WBC count and postoperative diagnosis results based on the 2018 ICM criteria. Group 0: Non-PJI group. Group 1: PJI group. Group 2: inconclusive groups. **B** The boxplot showing the association between synovial fluid PMN% and postoperative. **C** The boxplot showing the relationship between synovial fluid Alpha-defensin and postoperative diagnosis results based on the 2018 ICM criteria. **D** The association between synovial fluid Alpha-defensin and WBC count

Based on the postoperative 2018 ICM diagnostic results, the patients were divided into 3 groups: PJI group, Non-PJI group and inconclusive group. The levels of synovial fluid Alpha-defensin, WBC count and PMN% in the PJI group was significantly higher than that in the Non-PJI group and inconclusive group (Fig. 2A, B, C). Besides, the positive linear relationship was observed between log10 (alpha-defensin) and log10 (WBC count) (Fig. 2D, *r* ^2^=0.652). The details about the relationships were shown in Fig. 2.

### The ROC analysis of synovial fluid Alpha-defensin, WBC count, PMN% and LE strip test compared to 2018 ICM consensus

**Fig. 3:**
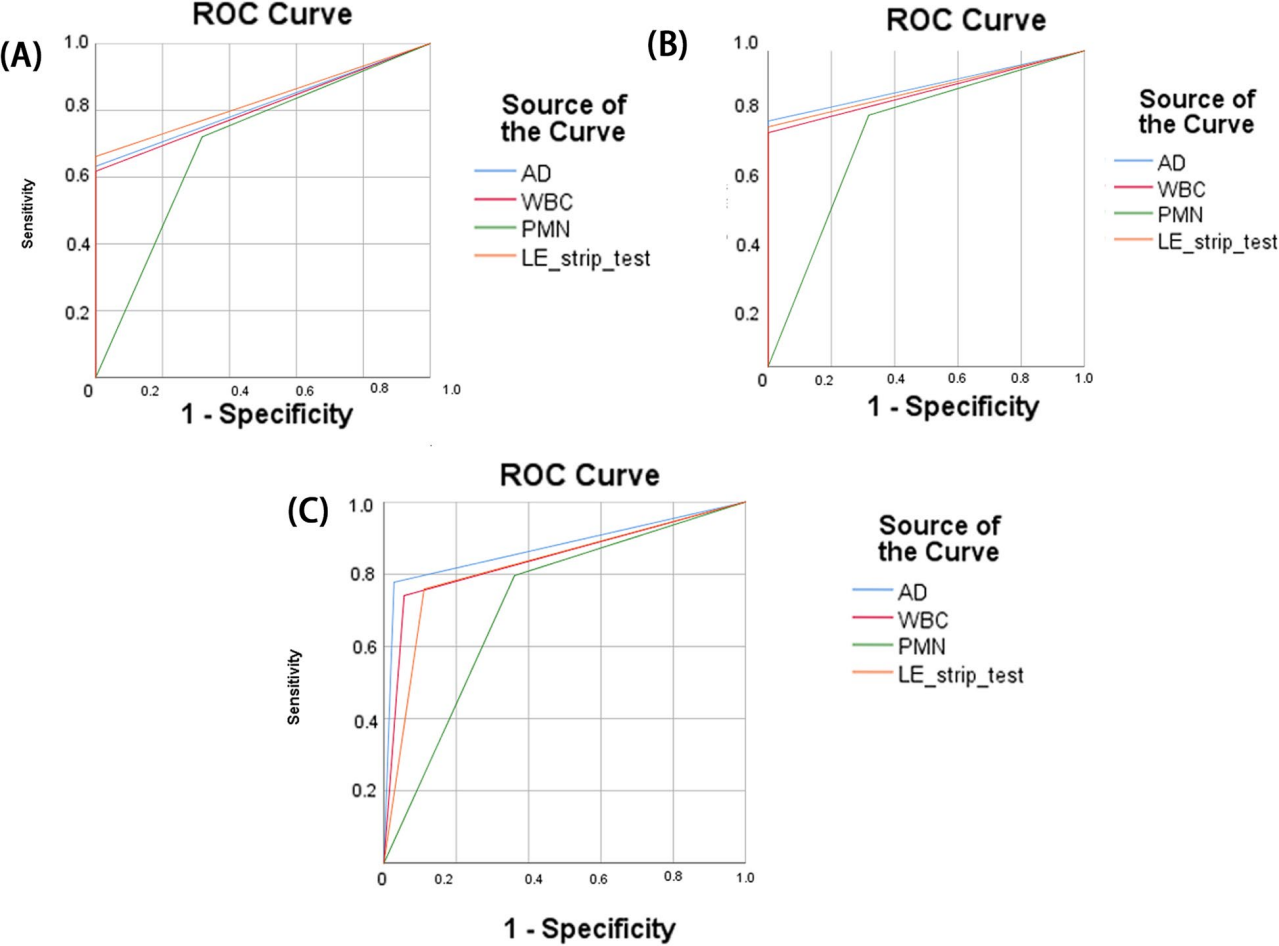
**A** The Roc curve of synovial fluid Alpha-defensin, white blood cell (WBC) count, PMN% and LE strip tests for PJI diagnosis in model 2. **B** The Roc curve of synovial fluid Alpha-defensin, white blood cell (WBC) count, PMN% and LE strip tests for PJI diagnosis in model 1. **C** The Roc curve of synovial fluid Alpha-defensin, white blood cell (WBC) count, PMN% and LE strip tests for PJI diagnosis in model 3

In model 1 and model 3 (Fig. 3B and C), Alpha-defensin test was the best single test for PJI diagnosis than synovial fluid WBC count, PMN% and LE strip test based on the 2018 ICM criteria (AUC: 0.889 (95% CI: (0.817,0.961)) and 0.875 95% CI: (0.800,0.950)). And in model 2 (Fig. 3A), synovial fluid alpha-defensin test and LE strip test show similar AUC for PJI diagnosis (0.816, 95% CI: (0.732,0.900) vs 0.813 95% CI: (0.750,0.912)). Besides, the sensitivity, specificity, positive predictive values (PPV), negative predictive values (NPV) and AUC of Alpha-defensin, WBC count, PMN% and LE strip tests was summarized in Table 4.

The details were shown in Table 5.

**Table 5:** The Sensitivity, specificity, PPV and NPV of synovial fluid WBC count, PMN%, LE strip test and Alpha-defensin test in comparison with postoperative PJI diagnostic results based on the 2018 ICM criteria

	Model	Alpha-defensin	WBC count	PMN%	LE strip tests
AUC (95%CI)	model1	0.889(0.817,0.961)	0.870(0.793,0.948)	0.739(0.609,0.869)	0.880(0.805,0.955)
	model2	0.816(0.732,0.900)	0.809(0.723,0.895)	0.701(0.572,0.830)	0.831(0.750,0.912)
	model3	0.875(0.800,0.950)	0.843(0.758,0.927)	0.718(0.606,0.830)	0.824(0.734,0.915)
Sensitivity (95%CI)	model1	0.78(0.64,0.87)	0.740(0.601,0.846)	0.796(0.661,0.889)	0.759(0.620,0.860)
	model2	0.632(0.506,0.743)	0.617(0.491,0.730)	0.721(0.597,0.819)	0.662(0.536,0.769)
	model3	0.778(0.640,0.875)	0.740(0.601,0.846)	0.796(0.661,0.889)	0.759(0.620,0.860)
Specificity (95%CI)	model1	1(0.815,1)	1(0.82,1)	0.682(0.451,0.853)	1 (0.815,1)
	model2	1(0.815,1)	1(0.815,1)	0.682(0.451,0.853)	1 (0.815,1)
	model3	0.972(0.838,0.998)	0.944(0.799,0.990)	0.639(0.462,0.787)	0.889(0.730,0.964)
NPV (95%CI)	model1	0.64(0.467,0.797)	0.611(0.44,0.76)	0.577(0.372,0.760)	0.628(0.449,0.780)
	model2	0.468(0.323,0.617)	0.458(0.316,0.606)	0.441(0.276,0.619)	0.489(0.339,0.640)
	model3	0.745(0.594,0.856)	0.708(0.557,0.826)	0.676(0.494,0.820)	0.711(0.554,0.832)
PPV (95%CI)	model1	1(0.896,1)	1(0.890,1)	0.860(0.726,0.937)	1(0.893,1)
	model2	1(0.898,1)	1(0.896,1)	0.875(0.753,0.944)	1(0.902,1)
	model3	0.976(0.862,0.998)	0.952(0.825,0.992)	0.768(0.632,0.867)	0.911(0.779,0.972)

*Model 1:* The inconclusive cases were excluded when the analysis was performed

*Model2:* The inconclusive cases were added into PJI group when the analysis was performed

*Model3:* The inconclusive cases were added into non-PJI group when the analysis was performed

### The sensitivity, specificity, positive predictive value (PPV) and negative predictive value (NPV) of synovial fluid Alpha-defensin, WBC count, PMN% and LE strip test compared to 2018 ICM consensus

Based on the Model 1, the sensitivity of synovial fluid Alpha-defensin, WBC count, PMN% and LE strip test was 78%,74%,79.6% and 75.9%, respectively. Based on the Model 2, the sensitivity of synovial fluid Alpha-defensin, WBC count, PMN% and LE strip test was 63.2%, 61.7%, 72.1% and 66.2%, respectively. Based on the Model 3, the sensitivity of synovial fluid Alpha-defensin, WBC count, PMN% and LE strip test was 77.8%, 74%, 79.6% and 75.9%, respectively. The detail about the sensitivity, specificity, PPV and NPV of these synovial fluid tests were summarized in Table 5.

## Discussion

The utility of Alpha-defensin in PJI diagnosis was controversial when conventional synovial fluid analysis was performed. Here, we performed a retrospective study to explore the utility of alpha-defensin in PJI diagnosis based on the 2018 ICM criteria [5]. We found that additional order of Alpha-defensin can’t change the both preoperative and postoperative PJI diagnostic results when synovial fluid analysis (WBC count, PMN% and LE strip test) was performed and the combination of synovial fluid WBC count, LE strip test and Alpha-defensin can’t change the diagnostic results compared to the combination of WBC count and LE strip test based on the 2018 ICM criteria. Besides, the additional order of Alpha-defensin can’t improve the consistency rate between preoperative diagnosis and postoperative diagnosis. And the direct cost-effectiveness was more than $8370 per case. Therefore, considering PJI diagnostic cost and the effectiveness of alpha-defensin tests, we recommended that the Alpha-defensin test was unnecessary when synovial fluid analysis tests (WBC count, PMN% and LE strip test) was ordered.

The use of Alpha-Defensin in PJI diagnosis is emerging in recent years because this test shows excellent sensitivity and specificity for PJI diagnosis and it was included in the PJI diagnosis panel [5, 6, 18]. However, some studies revealed that this test has comparable accuracy to synovial fluid white blood cell count, polymorphonuclear percentage and LE strip test for PJI diagnosis [7, 9, 11, 12]. Considering that the 2018 ICM criteria merged these three tests into the PJI diagnostic panel, one question raised–– whether all these three tests should be ordered for PJI diagnosis. The cost of Alpha-defensin test ($93 per case) was significantly higher than that of synovial fluid WBC count and LE strip test ($10 per case) and the clinical effect of alpha-defensin was unknown when the other two tests were performed [7, 11]. Here, we explored the risk–benefit ratio for additional order of alpha-defensin. When the 2018 ICM criteria were set as the “reference standard” for PJI diagnosis, no preoperative and intraoperative diagnostic results changed with the additional order of alpha-defensin test if both WBC count and LE test were performed (risk–benefit ratio: 90/0). Similarly, only one preoperative and intraoperative diagnostic result changed with the addition order of alpha-defensin test if only synovial fluid WBC count were performed (risk–benefit ratio: 89/1). Therefore, based on our results, we recommended the combination of synovial fluid analysis (WBC count, PMN%) and LE strip test as the synovial fluid markers to perform PJI diagnosis and the order of Alpha-defensin was unnecessary if the synovial fluid was performed and LE strip test was readable.

In this study, we found that the additional order of Alpha-defensin can’t change the PJI classification preoperatively and intraoperatively based on the 2018 ICM criteria. However, the additional order of LE strip test can change PJI classification based on the same criteria (8 inconclusive cases were identified as PJI or suspected infection). It indicated that Alpha-defensin can have similar diagnostic performance compared to the WBC in this diagnostic panel. Therefore, we explored the relationship between synovial fluid alpha-defensin and WBC count. We found that liner relationship was observed between log (AD) and log (WBC count) (Fig. 2, *r* ^2^ = 0.652). alpha-defensin was released by WBC to serve as a part of host-defence innate immune system and this characteristics of alpha-defensin can explain why there were linear relationship between log (AD) and log (WBC) and the use of synovial fluid alpha-defensin was unnecessary when synovial fluid analysis (WBC count and PMN%) were performed. And this significant linear correlation was also revealed in other studies [9].

When the 2018 ICM criteria were used to guide PJI diagnosis in clinical practice, the patients were divided into three groups inevitably: PJI group, Non-PJI group and inconclusive cases. We built three different models to handle the inconclusive cases and perform statistics analysis. For model 1, the AUC of these tests can be potentially overestimated because the inconclusive cases were excluded from the study. And the sensitivity and specificity of these tests in the “real world” can fall into the range calculated based on model 2 and model 3 because the inconclusive cases were classified into the PJI group and non-PJI group, accordingly. Besides, we also found that the levels of these tests in the inconclusive cases were lower than that in the PJI group but they were higher than that in the non-PJI group despite no significant difference was detected. It indicated that the levels of host inflammatory response in the inconclusive cases was between that in the PJI group and non-PJI group [18–20]. Septic or aseptic inflammation is hard to discern in these patients and the studies about these patients still need to be explored further.

There were still some limitations in this study. Firstly, the study was performed retrospectively and synovial fluid analysis (WBC count, PMN% and LE strip test) were ordered commonly in this study. However, synovial fluid analysis isn’t always feasible in clinical practice because of the existence of “dry joint” and “blood contamination”. Therefore, this study potentially excluded the patients with “dry joint” whose synovial fluid analysis was infeasible and the patients with “blood contamination” whose LE test wasn’t being readily available. This nature of design may add some bias to this research and the extension of our conclusion is questionable in special cases such as “dry joint”. Secondly, we applied identical WBC count and PMN% cutoffs for THA and TKA but some studies suggested that cutoffs may be different between the two types of joints. It may confound the results reported here. Thirdly, identifying pathogens is important for the diagnosis and treatment of PJI and “two identical positive clinical culture” were still the major criteria in PJI diagnosis with a high specificity. Culture negative PJI is estimated up to 20% and further molecular diagnostics such as sonication, PCR and next generation sequencing (NGS) were recommended when the PJI diagnosis was inconclusive, especially in culture-negative cases. However, considering that these diagnostic methods were not used as scoring criteria in the 2018 ICM criteria and there were still no widely accepted cut-off values and bioinformatic pipelines for metagenomic next generation sequencing (mNGS), prosthesis sonication and PCR tests, these molecular diagnostic tests weren’t further included and evaluated in this study. Finally, the study was performed retrospectively and the synovial fluid was stored at -80℃ until the Elisa test for Alpha-defensin test was performed.

## Conclusions

To conclude, Alpha-defensin assay exhibit high sensitivity and specificity for PJI detection as a standalone test based on the 2018 ICM criteria. However, the additional order of Alpha-defensin was unnecessary when corresponding synovial fluid analysis was performed (synovial fluid WBC count, PMN% and LE strip tests).

## Data Availability

All data and materials were in full compliance with the journal’s policy. And the data were obtained in Department of Orthopedic Surgery, The First Medical Center, Chinese PLA General Hospital. The datasets used and during the current study are available from the corresponding author on reasonable request.

## Abbreviations

TJA: Total joint arthroplasty
PJI: Periprosthetic joint infection
ICM: International consensus meeting
